# Antioxidant, antibacterial and antiviral effects of the combination of ginger and garlic extracts

**DOI:** 10.6026/973206300200011

**Published:** 2024-01-31

**Authors:** Saravanan Rajendrasozhan

**Affiliations:** 1 Department of Chemistry, College of Sciences, University of Ha'il, Ha'il 55473, Saudi Arabia

**Keywords:** Methanolic extract, DPPH, FRAP, antioxidant, antibacterial, antiviral

## Abstract

Garlic and ginger are well known as safe alternatives to traditional therapies. Limited information exists regarding antioxidant, antibacterial and antiviral capabilities of the combination of ginger and garlic. Standard methodologies were employed to determine the phytochemical compositions. Antioxidant activities were evaluated through DPPH and FRAP assays. Notably, in DPPH assay, combination of ginger and garlic extracts displayed significantly higher (85.44%, p < 0.005) antioxidant activity even at lower concentrations (6 mg/ml) compared to ginger and garlic extracts alone. Similar findings were observed for FRAP assay. At low concentration of extracts (25 µg/ml), combination of ginger and garlic exhibited significant (p < 0.005) increase in reducing activity (51%) compared to ginger or garlic extracts alone. Significant antibacterial and antiviral activities were exhibited by the combination of both ginger and garlic extracts as compared to ginger and garlic extracts alone. The combined effect of garlic and ginger exhibited a synergistic effect in bacterial and viral growth inhibition. These findings suggest that the diverse phytochemical compositions of the ginger and garlic varieties contribute to their strong antioxidant properties, potentially positioning them as valuable therapeutics for bacterial and viral infections. Further analysis will be required for their widespread utilization and pharmaceutical applications.

## Background:

Phenolic compounds constitute a substantial group of plant secondary metabolites found extensively across diverse higher plant organs, including vegetables, fruits, cereals, legumes, and beverages like tea, coffee, beer, and wine [1- 2]. They are found in all plant organs and are therefore an intrinsic part of the human diet [2]. Phenolic structures range from simple molecules like phenolic acids to extremely polymerized tannins [3]. When plants experience stress due to injury, their biological response triggers an accelerated production of phenols, contributing to the defence mechanism against injury, thereby aiding in wound healing and repair. Phenolic compounds are synthesized via the shikimic acid and phenylpropanoid pathways [4]. These compounds are involved in defence against ultraviolet radiation, parasites and predators, and contribute to the colours of plants [3]. According to Lin *et al.* they possess multifunctional roles in biological systems, functioning antioxidants, structural polymers, attractants, UV protectants, signal agents, and defence responders [4]. Phenolic compounds are also known for their antioxidant properties and confer health advantages by sustaining human physiological well-being, bolstering the body's resilience against oxidative harm, and mitigating the risk of ailments like cardiovascular diseases and cancer [5]. Studies have revealed that phenolic compounds impart various pharmacological effects, including metal ion chelation [6], antioxidant properties [2], vasodilation [7], anti-allergenic [8] and anti-inflammatory [9], antimicrobial [6,9, 10,11], and antitumor properties [12].

Metal ions have a crucial role in the survival and pathogenesis of viruses. They are intrinsic parts of viral proteins involved in various essential processes. Zinc (Zn^2+^), magnesium (Mg^2+^), and copper (Cu^2+^) are the predominant metal ions that bind with viral proteins. These metallic ions play pivotal roles in genome maturation, catalytic activation, reverse transcription, initial integration phases, and safeguarding new DNA copies. Additionally, metal ions are involved in the nucleocapsid protein-transactivation response and can induce conformational changes in viral proteins [13]. Furthermore, metal-induced oxidative stress has the potential to affect immune reactions against viruses, while imbalances in serum metal ion levels can disrupt the host's response to viral infections [14]. Overall, metal ions are essential for the structure and functionalities inherent to viruses, their survival tactics, and the development of disease within a host organism [13].

It has been established metals, such as Zn^2+^, Cu^2+^, Mg^2+^, and Mn^2+^, possess antiviral properties [13]. Zinc has been shown to have antiviral effects at high concentrations, with studies suggesting that free zinc may possess potent antiviral effects [15]. Copper and its alloys have also been identified as prospective materials in fighting viral infections, because of the ability of copper ions to inhibit virus's proteases and destroy the replication and propagation abilities of viruses [16]. Moreover, other biologically significant cations like Mn^2+^, have been found to inhibit the action of specific viral enzyme, such as human immunodeficiency virus (HIV) reverse transcriptase [6].

Similarly, metal ions play a crucial role in the virulence and viability of bacterial pathogens. Metal ions play an essential role in numerous physiological processes by acting as constituents within metallo-proteins, functioning as cofactors, or serving as structural components in enzymes [17-18]. Bacteria use specific uptake mechanisms to acquire essential metal ions such as iron, cobalt, nickel, copper, and zinc, which are required for their survival and pathogenesis [17,19]. Metal ions also play a role in signalling and regulation of virulence, and the maintenance of cellular metal ion homeostasis is crucial for bacterial viability [20]. Dysregulation of metal ion homeostasis can lead to bacterial pathogenesis and antibiotic resistance [17].

Garlic *(Allium sativum)* and ginger *(Zingiber officinale)* are acknowledged as safe alternatives to traditional therapies for a spectrum conditions, encompassing diabetes, hypertension, cardiac, neurological, inflammatory, renal, dental disorders, and specific forms of cancer [21,22,23, 24]. These two spices play a crucial role in traditional Asian culinary practices, thriving in favourable geographic and climatic conditions conducive to their cultivation. Medicinally significant plants and spices are routinely utilized in healthcare and veterinary applications across Asia. The compounds present in ginger and garlic, such as gingerol, shogaol, and allicin, are known for their broad-spectrum antibacterial activity [25]. Additionally, the metal content in garlic and ginger, including zinc, copper, iron, and manganese, may also play a role in their antibacterial activity [26]. The quality and quantity of plant extracts, along with their efficacy as antioxidant agents, typically impacted by several factors, including the attributes of the initial plant material, the extraction methodology employed, the extraction solvent used, and various other contributing factors [27]. Therefore, it is of interest to investigate the phytochemical composition, antioxidant potential and antimicrobial activity of ginger and garlic.

## Materials and Methods:

## Methanol based extractions:

Samples of ginger and garlic underwent extraction employing absolute (99.9%) methanol following the methodology detailed by Antolovich *et al.* [30]. Each 100 ml amber bottle contained 2 g of freshly grated ginger and garlic, combined with 30 ml of a designated solvent. The organic blends were agitated for 1 hour at 300 rpm using a mechanical shaker and subsequently shielded from light for 72 hours to prevent potential reactions induced by light exposure. Following this period, the extracts were filtered using Whatman filter paper No. 1 and utilized for the assays.

## Total phenolic and flavonoid content

The total phenolic content in garlic and ginger aqueous extracts was determined using a colorimetric method developed by Çayan *et al.* [28]. This method involved combining diluted Folin-Ciocalteu reagent and a 7.5% Na_2_CO_3_ solution with the extract in a microplate well. After a 30-minute incubation at 25°C in darkness, the optical density (OD) was measured at 765 nm using a FLUOstar Omega micro-plate reader. Gallic acid served as the standard reference, and the results were quantified as milligrams of Gallic Acid Equivalent (GAE) per gram of dry extract. Triplicate measurements were conducted to ensure result precision and accuracy, shedding light on the phenolic composition critical for understanding the health-related properties of these extracts. For the determination of flavonoid content, a modified method from Zhishen *et al.* [29] was employed. This involved analysing 100 µL of the extract with a specific sequence of solutions (Mixture A and B) and incubation periods. After successive additions of NaNO_2_, anhydrous aluminum chloride (AlCl_3_), and NaOH solutions, the resulting mixture was evaluated for absorbance at 496 nm, using the same microplate reader. The findings were expressed as milligrams of Quercetin Equivalent (QE) per gram of the sample (mg QE/g) and were obtained through triplicate analyses, ensuring accuracy, and minimizing experimental error. This method provided a precise assessment of flavonoid content, crucial for understanding the health-promoting potential of garlic and ginger extracts.

## Antioxidant assays of ginger and garlic extracts:

## DPPH assay:

The antioxidant potential of aqueous extracts from ginger and garlic was methodically assessed using the 2,2-diphenyl-1-picryl hydrazyl (DPPH) radical assay, with slight alterations to the established protocol [30]. DPPH, recognized for its vivid purple hue in solution, transforms to a colourless or faint yellow shade upon interaction with antioxidants, a change measurable via spectrophotometry at 517 nm. This assay, corroborated by previous studies [31], accurately gauges antioxidant capabilities. The experiment involved preparing varying concentrations (ranging from 0 to 10 mg/ml) of ginger and garlic extracts in analytical-grade methanol, facilitating a comprehensive assessment across concentration gradients. Vitamin C served as the benchmark antioxidant for comparison.

The procedure entailed mixing the extracts with DPPH in methanol, with a control sample of solely methanol and DPPH. After a five-minute incubation, absorbance readings at 517 nm were taken using a spectrophotometer. The calculated radical scavenging activity, expressed as a percentage, indicated the extracts' ability to counter DPPH radicals. Higher percentages denote heightened antioxidant potency. Insights into the antioxidant capacities of ginger and garlic extracts were gained by conducting this assay in triplicate. These findings significantly contribute to comprehending the potential health advantages associated with these natural extracts.

% inhibition of DPPH = (AB-AA)/AB*100

AB - absorbance of control sample; AA - absorbance of tested extract solution

The outcomes were presented in two forms: as the percentage inhibition of DPPH and the determination of minimum inhibitory concentrations (IC_50_). IC_50_ signifies the concentration necessary to reduce DPPH by 50%, wherein lower values denote more robust antioxidant activity.

## FRAP assay:

The ferric reducing antioxidant power (FRAP) was employed to detect antioxidant activities of both ginger and garlic extracts [32]. Ascorbic acid, 1ml (0-100 µg/mL) or ginger or garlic extracts (0-100 µg/mL) were added to 2.5 mL phosphate buffer (0.1 M, pH 6.6) then these were mixed with 1% potassium ferricyanide (2.5 mL). Incubate the mixture for 15 min at 50°C. Then trichloroacetic acid (2.5ml, 10%) was added stop the reaction. Then centrifuged the mixtures at 2500 rpm for 15 min and collect the supernatant. Add ferric chloride solution (0.5 mL, 0.5%) to the collected supernatant (2.5 mL) and distilled water mixture (2.5 mL). Absorbance was analysed at 700 nm. Ascorbic acid was served as standard and a standard curve was made and followed Beer's Law and the regression coefficient was calculated as 0.9982 and a slope of 0.0039. The standard curve's equation is y = 0.0039x + 0.0209.

Percent reducing power = A_control_ - A_sample_/A_control_ x 100

A_control_ = Control sample absorbance

A_sample_ = Sample absorbance

## Antibacterial activity:

The antibacterial efficacy of individual extracts was assessed against a spectrum of four bacterial strains: Pseudomonas aeruginosa (ATCC 39327), Staphylococcus aureus (ATCC BAA-41), *Mycobacterium tuberculosis* (ATCC 27294), *Escherichia coli* (ATCC 11775), and Psuedomonas fluorescence (ATCC 13525). Determination of the minimum inhibitory concentration (MIC) for each extract against these bacteria was carried out using the broth microdilution technique. The MIC determination involved creating stock solutions of each ginger garlic extract in trypticase soy broth (TSB), spanning concentrations from 0 to 35 mg/mL.

The process included combining 5 µL of bacterial inoculum (1x 108 CFU/mL) with 295 µL of various extract dilutions in 96-well microplates. Positive controls (inoculum without extract) and negative controls (extract without inoculum) were incorporated. Following a 24-hour incubation, at 37°C, the MIC values were identified as the lowest concentrations that effectively impeded bacterial growth, confirmed by viable bacterial counts on agar plates.

Moreover, the study monitored the growth trajectories of the tested bacteria (initial inoculum of 1x 10_8_ CFU/mL) exposed to the fungal extracts under identical experimental conditions. Incubation of the microplates at 37°C for 16 hours with intermittent shaking allowed for the recording of optical density (OD) readings at 600 nm every 30 minutes. The experiment underwent replication three times. Analysis of the growth data utilized the Baranyi function, with the derivation of crucial parameters such as adaptation or lag time (λ; h), maximum growth rate (µ_max_; OD/h), and maximum growth Y_max_ (Y_max_; OD) for each growth curve [33].

## Antiviral activity of extracts:

The antiviral potential of the ginger and garlic extracts was evaluated against bacteriophages DS6A (ATCC 25618-B2), T4 (ATCC 11303-B4), phi-S1 (ATCC 27663-B1), CDC-47 (ATCC 27691-B1), and bacteriophage 2 (ATCC 14203-B1). Each viral suspension, containing 2 logs of plaque-forming units (PFU/mL), was exposed to a concentration of 0.9 mg/mL of the corresponding extract [34]. These mixtures underwent thorough agitation for 10 minutes, alongside a control mixture containing untreated virus for comparative analysis. Next, viral infection quantification utilized the double agar layer method. Both treated and controlled viruses were introduced into bacterial hosts- T4 for *Escherichia coli* strain (ATCC 11303), DS6A for *M. tuberculosis* strain (ATCC 25618), psi-S1 for *P. fluorescens* (ATCC 27663), bacteriophage 2 for *P. aeruginosa* Migula (ATCC 14203), and CDC-47 for *S. aureus* (ATCC 27691). Initially, these blends were combined with a top layer comprising melted agar (consisting of 3% tryptic soy broth, 0.5% NaCl, and 0.6% agar), which was subsequently poured onto a solid agar bottom layer (comprising 3% tryptic soy broth, 0.5% NaCl, and 1.2% agar). Following solidification, the plates were incubated for 24 hours at 37°C. After incubation, PFU counts in both the treated and control groups were determined, and the percentage of antiviral activity was computed by subtracting the titer values of the treated samples from those of the untreated control. To ensure robustness and consistency, this experiment was replicated three times.

## Statistical analyses:

Prism (Graphpad) software (trial version) was used for statistical analysis. P-values less than 0.05 exhibited significance.

## Results and Discussion:

Compounds present in ginger and garlic are also known for their antioxidant properties and have been found to have advantages of sustaining human health, bolstering the body's resilience against oxidative harm, and mitigating the onset of diseases like cardiovascular diseases and cancer [5,35].

## Phenolic and flavonoid contents in extracts:

The aqueous solvent extracts of garlic and ginger exhibited a phenolic content of 22.59 ± 0.022 and 31.59 ± 0.027 mg GAE/100 g, respectively (Table 1). Notably, a significantly (p > 0.05), higher phenolic content was observed in ginger compared to garlic. The phenolic content in both extracts closely aligned with previous study findings, indicating consistency in their levels of GAE/100 g [22,27]. These observations underscore the impact of different forms on the overall phenolic content. Ginger has been identified as having elevated concentrations of gingerols, shogaols, and paradols, constituting the predominant polyphenols present in fresh ginger. The presence of hydroxyl (OH) groups within the chemical structure of phenolic compounds contributes to their antioxidant potential [22]. Methanolic extracts derived from ginger samples have demonstrated elevated phenolic content, whereas aqueous extracts obtained from garlic samples have exhibited heightened phenolic content. The occurrence of fat-soluble phenolic compounds, including hydroxybenzoic acid, within methanolic extracts of garlic could potentially contribute to the observed elevation in overall phenolic content [36]. Thus, total phenolic content serves as a screening parameter for antioxidant activity in plants [37].

Flavonoids represent a class of polyphenolic secondary metabolites characterized by their low molecular weight, ubiquitously present across plant species. Flavonone, flavone, isoflavone, flavonol, catechin, naringin, and anthocyanins are among the distinct classes of flavonoids [38]. In this study, both garlic and ginger showcased appreciable levels of flavonoid compounds, measuring 17.96 ± 0.026 and 24.58 ± 0.033 QE/100 g, respectively (Table 1). Ginger notably exhibited a significantly higher (p < 0.05) quantity of flavonoids compared to garlic. These findings align consistently with previous studies [22,25, 27]. Methanolic extracts derived from ginger have shown a higher concentration of flavonoids in comparison to aqueous extracts obtained from garlic [22]. The discernible contrast in flavonoid content between ginger and garlic can be ascribed to variances in their chemical compositions and the methodologies employed during the extraction process.

## Antioxidant activity of ginger and garlic extracts:

**Figure 1:**
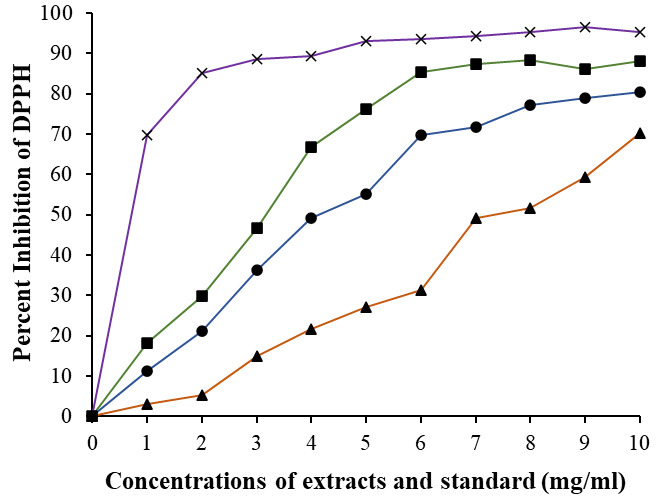
DPPH free radical scavenging ability of ginger (-●-) and garlic (-▲-), and combination of both ginger and garlic extract (-■-) extracts in varying concentration. Standard was selected as vitamin C (-x-). Samples were conducted in triplicates and values are given in mean. Comparison between ginger/garlic vs combined was performed based on t test, and significance was defined as p < 0.05.

The free radical scavenging potential of garlic and ginger extracts was assessed via the DPPH assay as shown in Figure 1. Results indicated a dose-dependent increase in free radical scavenging activity for both extracts, ranging from 1 to 10 mg/ml. A comparison was drawn between the inhibition potentials of locally utilized ginger and garlic extracts. A standard graph was constructed using vitamin C as a benchmark. At a concentration of 10 mg/ml, the garlic and ginger extracts exhibited 71.14% and 89.49% DPPH oxidation inhibition, respectively. A significant discrepancy was noted in the percentage of DPPH oxidation between ginger and garlic across varied concentrations (1 - 10 mg/ml). Other studies have demonstrated heightened oxidation activities in ginger and garlic organic extracts [22,39, 40]. The heightened presence of phenolic compounds in ginger potentially contributes to its superior antioxidant activity and capacity for scavenging free radicals when compared to garlic [36]. Furthermore, combination of both exhibited significant increase (85.44%; p < 0.005) in antioxidant activity even at lower concentrations (6 mg/ml) as given in Figure 1.

Furthermore, IC_50_ values were computed for both ginger and garlic extracts for DPPH assay. Ginger showcased an IC_50_ value of 4.13 mg/ml, significantly lower (p < 0.01) than garlic's IC_50_ value of 6.3 mg/ml. These findings suggest a notably stronger oxidation activity in ginger compared to garlic [22,41, 42]. Therefore, the discrepancy in the IC_50_ values between ginger and garlic is linked to the differences in their phytochemical profiles, particularly their phenolic and flavonoid contents, which ultimately affect their antioxidant capacities. When extracts from both were mixed together showed further decreased in IC_50_ value 2.89 mg/ml.

**Figure 2:**
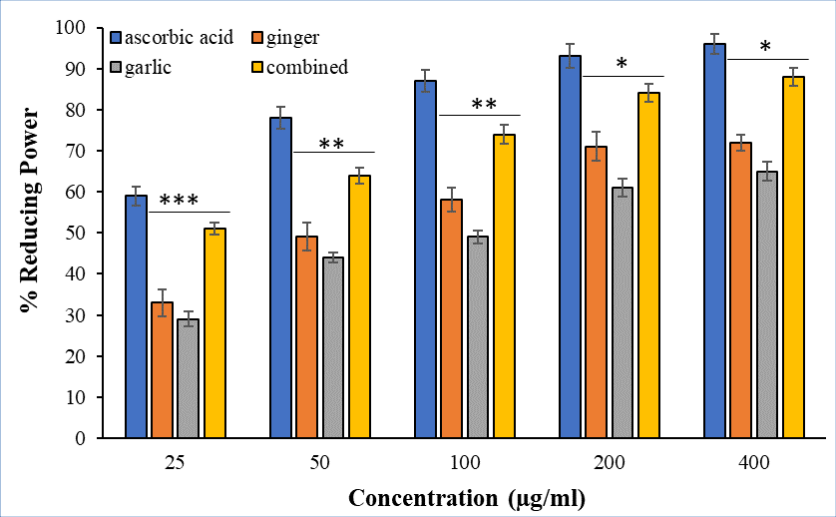
FRAP assay was conducted using ascorbic acid as standard. Ginger, garlic extracts, and their combination were analysed at concentrations (25-400 µg/mL). Each sample was run in triplicate and presented as means ± SEM. Comparison between two groups was performed based on t test, and significance was defined as p < 0.05. Signs *, **, and *** represent p values 0.05, 0.01, and 0.005 respectively.

FRAP assay was employed to evaluate the reducing capability of ginger, garlic extracts and their combination. In this test there is a reduction of Fe^+3^ to Fe^+2^ with the help of the extracts. At lower concentration (25 µg/ml) of extracts, calculations showed ginger and garlic exhibited 33% and 29% of reducing activities, respectively. And a combination of both extracts showed a significantly higher (p < 0.005) reducing power (51%). Reducing powers for ginger, garlic and combination of both increases with increasing concentrations (50, 100, 200, and 400 µg/ml) of extracts. However, with higher concentrations the p values comparing between ginger/garlic alone and combination of both were decreased as given in Figure 2. This result exhibited an improved ferric reducing function when both ginger and garlic extracts were mixed.

## Antibacterial activity of ginger and garlic extracts:

Table 2 presents the antibacterial effects of the extracts. Among the bacteria tested, the ginger extract exhibited the lowest minimum inhibitory concentration against *L. monocytogenes* (11 mg/ml), followed by *E. coli,* *Mycobacterium tuberculosis*, and *S. aureus*, showcasing a similar trend in the garlic extract. However, the MIC values for garlic were higher across all bacteria. Notably, *L. monocytogenes* displayed higher sensitivity to the extracts (17 mg/ml), while S. enterica demonstrated greater resistance to both extracts. Interestingly, no inhibitory activity was observed against *S. aureus* at the tested concentrations (0-41 mg/mL). This outcome correlates with the higher presence of phenolic and flavonoid compounds in ginger in comparison to garlic, known for their antibacterial properties [43- 44]. Previous finding also suggested the antibacterial effectiveness of garlic and ginger against multidrug-resistant *Escherichia coli* (MDR E. coli). [45]. The combined effect of garlic and ginger exhibited a synergistic effect in bacterial growth inhibition (Table 2) and the maximum effects were observed against *Mycobacterium tuberculosis* (p < 0.01) and E. coli (p < 0.05). Ginger and garlic are known to contain various phenolic compounds that exhibit antibacterial properties, leading to their death. Therefore, the phenolic content of ginger and garlic is believed to contribute to their antibacterial properties and potential use as natural alternatives to conventional antibiotics.

## Antiviral activity:

Methanolic extracts of both garlic and ginger exhibited antiviral activities for the bacteriophages selected in this assay. Garlic showed maximum activity against phi-S1 and ginger showed for DS6A. The combined effect of both was observed in all bacteriophages except bacteriophage CDC-47 as shown in Table 3. Bacteriophage 2 showed a significant antiviral activity between garlic compared with combination. Furthermore, significant effects were observed in DS6A, T4 and phi-S1 when combination of both garlic and ginger was utilized, exhibiting evident antiviral activities. Bioactive compounds in ginger and garlic extracts may interfere with the bacteriophage-bacteria interaction, inhibiting the multiplication of bacteriophages and reducing their titers [45]. Furthermore, the antibacterial effects of these extracts might indirectly support their antiviral activity since healthier bacteria, less susceptible to bacteriophage infection due to these extracts, could potentially limit the viral infection [46].

## Conclusion:

The study concluded that both ginger and garlic extracts boast substantial quantities of phenolic compounds. Generally, ginger extracts exhibited higher overall levels of total phenolics, flavonoids, and vitamin C compared to garlic. The use of an organic solvent proved more effective than water in extracting phytochemicals. However, water demonstrated greater efficiency in extracting vitamin C when compared to methanol. The antioxidant potential of the extracts, evaluated through DPPH assays, displayed a strong correlation with their total phenolic and flavonoid content. Notably, ginger extracts showcased elevated levels of total phenolic content and displayed robust antioxidant activities. To further advance their utilization as natural antioxidants, it is recommended to quantify the specific phenolic acids and flavonoids present in ginger and garlic extracts.

## Figures and Tables

**Table 1:** Total phenolic and flavonoid contents in garlic and ginger methanolic extracts.

**Preliminary screening**	**Garlic extract**	**Ginger extract**
Dry powder (g)	50	50
Percent yield (%)	5.19	5.93
Extract (methanolic)	yes	Yes
Total phenolic compounds (mg GA equivalent/g dry weight of extract)	22.59 ±0.022	31.59 ± 0.027*
Total flavonoid content (mg quercetin equivalent/g dry weight of the extract)	17.96 ± 0.026	24.58 ± 0.033*
Symbol* represents the significant change (p < 0.05). Gallic acid and of quercetin were used to prepared standards graphs.

**Table 2:** Minimum inhibitory concentrations (MIC) of garlic and ginger extracts against pathogenic bacteria

**Bacteria**	**Minimum Inhibition Concentration (mg/ml)**		
	**Garlic**	**Ginger**	**Garlic & Ginger**
*M. tuberculosis*	27	20	13**
*E. coli*	19	14	8*
*S. aureus*	47	41	37
*P. aeruginosa*	26	22	18
*P. fluorescence*	17	11	9

**Table 3:** Antiviral activity of ginger and garlic extracts.

**Bacteriophage**	**Target**	**Percent Plague Inhibition**		
		**Garlic**	**Ginger**	**Garlic & Ginger**
DS6A	*M. tuberculosis*	11.51	17.69	27.33*
T4	*E. coli*	9.47	14.19	19.23*
CDC 47	*S. aureus*	12.9	13.6	14.9
2	*P. aeruginosa*	13.2	17.9	21.41#
*phi-S1*	*P. fluorescence*	13.55	15.13	31.79**
Sign# represents significance comparison between either ginger or garlic and combination.
Sign* represents significance between ginger/garlic and combination.
